# Root Bark Extract of *Oroxylum indicum* Vent. Inhibits Solid and Ascites Tumors and Prevents the Development of DMBA-Induced Skin Papilloma Formation

**DOI:** 10.3390/molecules27238459

**Published:** 2022-12-02

**Authors:** Seema Menon, Jawaher J. Albaqami, Hamida Hamdi, Lincy Lawrence, Menon Kunnathully Divya, Liya Antony, Jose Padikkala, Shaji E. Mathew, Arunaksharan Narayanankutty

**Affiliations:** 1 Department of Biochemistry and Plant Biotechnology, Amala Cancer Research Centre, Amala Nagar, Thrissur 680555, India; 2 Department of Zoology, Kodungallur Kunjikuttan Thampuran Memorial Government College, Pullut, Kodungallur, Thrissur 680663, India; 3 Department of Biology, College of Science, Taif University, P.O. Box 11099, Taif 21944, Saudi Arabia; 4 Zoology Department, Faculty of Science, Cairo University, Giza 12613, Egypt; 5 Division of Cell and Molecular Biology, PG & Research Department of Zoology, St. Joseph’s College (Autonomous), Devagiri, Calicut 673008, India

**Keywords:** *Oroxylum indicum*, antioxidant activity, dimethyl benz(a) anthracene, skin papilloma, antitumor activity

## Abstract

*Oroxylum indicum* is a traditionally used plant in Ayurvedic and folk medicines. The plant is useful for the management of gastrointestinal diseases as well as skin diseases. In the present study, we analyzed the antitumor potential of *O. indicum* in Dalton’s lymphoma ascites tumor cells (DLA) and Ehrlich ascites carcinoma (EAC)-induced solid and ascites tumors. Further, the potential of *O. indicum* extract (OIM) on skin papilloma induction by dimethyl benz(a) anthracene (DMBA) and croton oil was evaluated. The chemical composition of the extract was analyzed using UPLC-Q-TOF-MS. The predominant compounds present in the extract were demethoxycentaureidin 7-*O*-rutinoside, isorhamnetin-3-*O*-rutinoside, baicalein-7-*O*-glucuronide, 5,6,7-trihydroxyflavone, 3-Hydroxy-3′,4′,5′-trimethoxyflavone, 5,7-dihydroxy-3-(4-methoxyphenyl) chromen-4-one, and 4′-Hydroxy-5,7-dimethoxyflavanone. Treatment with high-dose OIM enhanced the percentage of survival in ascites tumor-bearing mice by 34.97%. Likewise, high and low doses of OIM reduced the tumor volume in mice by 61.84% and 54.21%, respectively. Further, the skin papilloma formation was brought down by the administration of low- and high-dose groups of OIM (by 67.51% and 75.63%). Overall, the study concludes that the *Oroxylum indicum* root bark extract is a potentially active antitumor and anticancer agent.

## 1. Introduction

Cancer is characterized by the abnormal and uncontrolled proliferation of cells, which possess the ability to metastasize or invade other parts of the body [1,2]. The invading cells form a subset of neoplasms, which either form a tumor lump or a diffusely distributed cell population [3,4]. Chemotherapy is one of the common modalities of cancer treatment. Chemotherapeutic agents eliminate the rapidly proliferating cells, including hematopoietic bone marrow cells, hair follicles, digestive tract epithelial cells, and reproductive tract cells, apart from the cancer cells, which essentially are the prime targets [5]. This poses serious side effects that affect vital organs, such as the heart, lungs, kidneys, and digestive organs [6]. Clinically used chemotherapeutics are also facing issues of resistance by various molecular pathways [7,8,9]. Hence, there is a need for novel anticancer drug candidates to overcome the issues associated with present-day chemotherapeutics [10].

*Oroxylum indicum* Vent. is one of the ten plants whose roots are used as an ingredient of the *Dashamoola* combination in Ayurveda [11,12]. Different parts of the tree also find use in folklore medicine to cure ailments such as urinary infections, bronchitis, leukoderma, diarrhea, nasopharyngeal cancers, oral cancers, etc., as reviewed by Deka, et al. [13], Mao [14], and Preety and Sharma [15]. The root bark possesses anti-ulcer [16], immunomodulatory [17], antioxidant [18], and hepatoprotective [19] properties. The antiproliferative potential of different fractions of the root bark has also been reported [20] in human breast carcinoma cells. Still, thorough research on its anticancer properties in in vivo models has not been conducted.

Animals, including mouse models, reiterate the human equivalent of different malignancies [21]. Anticancer agents of plant origin bring about the inhibition/suppression of carcinogenesis, thereby acting as chemopreventive agents, or they pose toxicity to the cells in already developed tumors, thereby reducing tumor burden [22,23]. Ehrlich ascites carcinoma (EAC) is a rapidly proliferating, experimental transplantable tumor maintained in outbred mice by a series of intraperitoneal passages. It was originally identified as a murine mammary adenocarcinoma, which later was adapted to ascites form [24,25], and has been exploited for many chemotherapeutic studies [26,27,28]. The antitumor activity of any agent against Ehrlich ascites can be assessed by cytological examination of the ascites cells post-treatment, calculating the increase in survival time and/or by measuring the number of ascites formed after treatment [24]. Our study relied on the evaluation of the antitumor activity of *Oroxylum indicum* Vent. root bark, in EAC cells, in terms of its effect on the increase in the average life span.

Earlier models of evaluation of antitumor activity included murine models induced with ascitic leukemia using different types of cancer cell lines [29,30], but were not adequate enough for the identification of therapeutic agents against solid tumors [31,32]. In our study, the antitumor efficacy of the extract was evaluated using the solid tumor model in mice, inoculated intramuscularly with Dalton’s lymphoma ascites (DLA) cell line, which has been known to be in use for many similar investigations before [33,34]. The 7, 12-dimethyl-benz[a] anthracene (DMBA) acts as an inducer of the two-step mutagenesis and the TPA present in croton oil promotes the malignant conversion of mouse skin tumors [35]. Overall, the present study evaluates the antitumor and anticarcinogenic potential of *O. indicum* Vent. root bark in multiple models in mice.

## 2. Results

### 2.1. Phytochemical Analysis

#### 2.1.1. Qualitative Phytochemical Analysis

**Figure 1:**
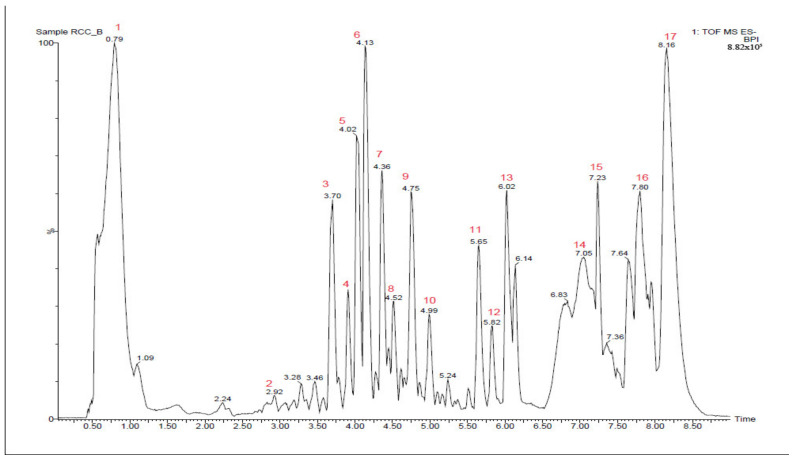
Total ion chromatogram of *O. indicum* Vent. wild root bark (OIM) extract subjected to UPLC-Q-TOF-MS analysis. The compounds corresponding to the peak numbers are listed in Table 1.

Figure 1 shows the total ion chromatogram of the OIM extract subjected to UPLC-Q-TOF-MS analysis. The compounds provisionally identified (based on previous fragmentation data from the literature) are enlisted in Table 1.

#### 2.1.2. HPLC-Based Quantification of Chrysin and Baicalein

The quantitative determination of chrysin and baicalein by HPLC is shown in the chromatogram (Supplementary Figure S1). The quantity of baicalein was 1.794 ± 0.23 mg/g of *O. indicum* powder. The quantity of chrysin was 0.725 ± 0.08 mg/g of the powder of *O. indicum* (Table 2).

### 2.2. Cytotoxicity of O. indicum Extract

**Figure 2:**
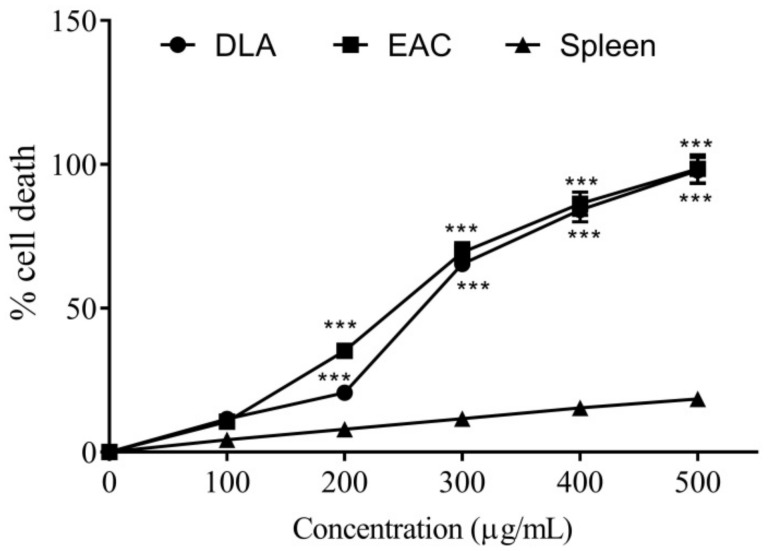
In vitro cytotoxicity of different concentrations of methanolic extract of *O. indicum* Vent. root bark (OIM) on DLA and EAC cell lines and normal spleen cells. *** Indicates a significant variation with *p* < 0.001 with the cytotoxicity in spleen cells.

The methanolic extract of *Oroxylum indicum* Vent. showed a profound decline in the viability of DLA and EAC cell lines in a dose-dependent pattern (Figure 2). No cell death was observed in the control. With OIM extract concentrations at 259.07 and 252.53 µg/mL respectively, 50% death of DLA and EAC cells occurred. The extract was found to be non-toxic to spleen cells up to the highest concentration used (500 µg/mL).

### 2.3. Effect of OIM Extract on Ascites Tumor

**Figure 3:**
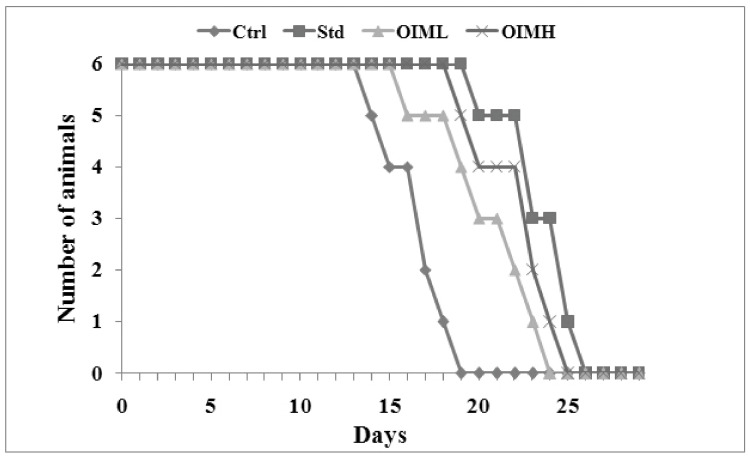
Effect of methanolic extract of *O. indicum* Vent. (OIM) on the average survival period of ascites tumor-bearing animals. Ctrl—untreated negative control; Std—positive control, treated with cyclophosphamide (i.p.) at 10 mg/kg b.wt.; OIML—low-dose group treated with OIM at dosage 200 mg/kg, and OIMH—high-dose group treated with OIM at dosage 400 mg/kg b.wt.

All animals developed ascites tumors upon receiving intraperitoneal inoculation of EAC cells. When the untreated control group sustained only an average survival period of 16.67 ± 1.86 days, treatment with OIM extract at 400 and 200 mg/kg increased the average life span to 22.5 ± 2.43 (*p* < 0.01) and 20.67 ± 2.94 (*p* < 0.05) days, respectively. OIMH treatment elevated the percentage increase of survival by 34.97%. The animals under standard drug cyclophosphamide survived for 23.67 ± 2.16 days (percentage increase in survival—41.99%) (Figure 3).

### 2.4. Effect of OIM Extract on Solid Tumor

**Figure 4:**
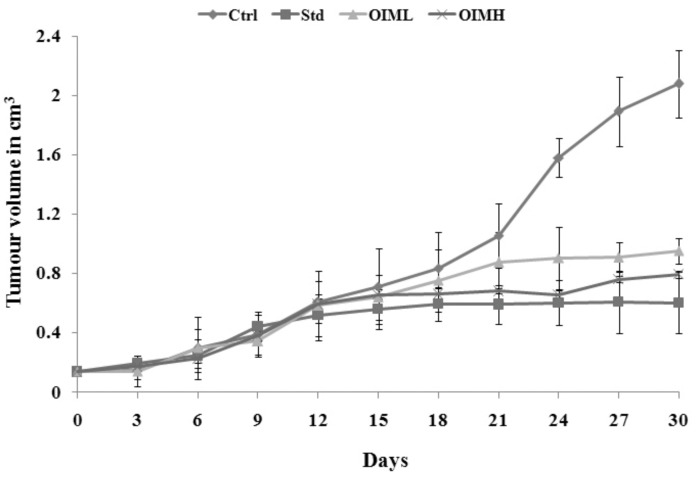
Effect of methanolic extract of *O. indicum* Vent. (OIM) on tumor volume in solid tumor-bearing animals. Values are expressed as mean ± SD for 6 animals. Ctrl—untreated negative control; Std—positive control, treated with cyclophosphamide (i.p.) at 10 mg/kg b.wt; OIML—low-dose group treated with OIM at dosage 200 mg/kg b.wt., and OIMH—high-dose group treated with OIM at dosage 400 mg/kg b.wt.

All animals who received intramuscular DLA injection developed tumors by the sixth day of induction, which grew in volume in successive days. Yet, compared to the final volume recorded on the 30th day in the control group (2.07 ± 0.23 cm^3^), *O. indicum Vent.* extract-treated groups showed a marked decrease (*p* < 0.01) in tumor volume up to 0.79 ± 0.03 cm^3^ in OIMH and 0.95 ± 0.09 cm^3^ in OIML. OIMH and OIML treatment brought about 61.84% and 54.21% inhibition of tumor growth, respectively, closer to that affected by the cyclophosphamide treatment in the standard animal group (71.05%) (Figure 4).

### 2.5. Effect of OIM Extract on Hematological Parameters

**Figure 5:**
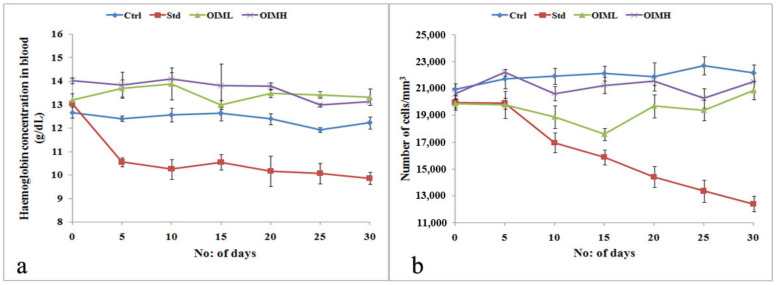
Comparison of hematological parameters between cyclophosphamide-treated animals and the other study groups during solid tumor induction. (**a**) Percentage hemoglobin (g/dL) and (**b**) total leucocyte count (no: of cells/mm^3^) of Ctrl—tumor-induced group, having received no treatment; Std—cyclophosphamide (i.p.) treated group at dosage 10 mg/kg b.wt; OIML—low-dose group treated with OIM at dosage 200 mg/kg b.wt., and OIMH—high-dose group treated with OIM at dosage 400 mg/kg b.wt.

Though it was evident that animals treated with cyclophosphamide exhibited substantial anti-tumor activity in solid tumor models, there was a gradual reduction in the hemoglobin concentration (Figure 5a) and total leucocyte count of the animals, which was not obviously seen in the control, OIML, or OIMH groups (Figure 5b).

### 2.6. Effect of OIM Extract on Skin Papilloma

**Figure 6:**
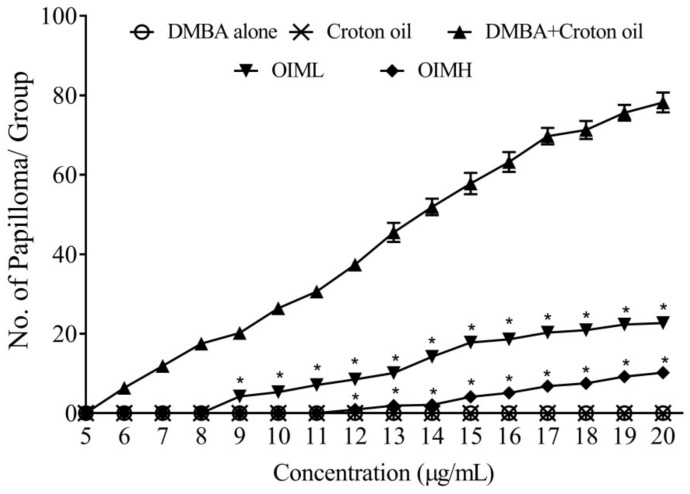
Effect of methanolic extract of *O. indicum* (OIM) on skin tumorigenesis in the DMBA–croton oil-induced animals. Group I: animals induced with 470 nmol of DMBA alone, in 200 acetone; Group II: animals induced 1% croton oil alone, in 200 acetone; Group III: animals induced with DMBA plus croton oil, which served as negative control; Group IV: [(DMBA + croton oil + OIM 5% in 200 μL distilled water)]; and Group V: [(DMBA + croton oil + OIM 10% in 200 μL distilled water)]. * indicate significant variation at *p* < 0.05 with DMBA + croton oil group.

The application of either DMBA or croton oil alone did not induce papilloma in any of the animals. Meanwhile, DMBA plus croton oil application in untreated control animals led to full-blown papilloma development (100% incidence). However, OIM application at low and high regimes reduced the incidence of a tumor to 83.3 and 66.6%, respectively (Table 1). Papilloma onset was registered in the control group in the 6th week of the topical application itself, whereas it was delayed in treated groups—9th week in OIML and 12th week in OIMH (Figure 6). Table 1 shows that the average number of papillomas per animal was significantly (*p* < 0.01) lower in treated mice compared to the control. OIM treatment was found to inhibit papilloma development in low- and high-dose groups by 67.51% and 75.63%, respectively.

## 3. Discussion

Cell-based screening assays are widely employed in drug discovery systems for determining the anticancer properties of both synthetic and plant-derived compounds, and the viability measurements of cancer cell lines in the presence of the tested compound are preliminary steps in drug screening. In the study, we found significant in vitro anticancer properties of OIM extract using short-term cytotoxicity assays in DLA and EAC cell lines, where non-toxic effects were noticed in normal splenocytes. Methanolic and aqueous extracts of *O. indicum* bark were previously demonstrated to induce cytotoxicity in MDA-MB-435S cell lines, as determined by the XTT assay [36]. The anti-proliferative ability of the methanolic extracts of fruits from *O. indicum* was studied on HL-60 cell lines, with baicalein identified as an active principle in the extract [37]. The flavonoid (already reported to be present in the root bark [38]) may be responsible for its capability to induce mortality in DLA and EAC cell lines, as uncovered by the present study. 

Followingly, the antitumor activity of *O. indicum* Vent. root bark extract was evaluated using EAC-induced ascites tumor models. Prolongation of the life span of animals is a reliable criterion for adjudging an anticancer agent. Though death is inevitable, OIM in high doses showed a significant increase in the survival rate of mice, compared to untreated control. However, OIM extract at a low dose (200 mg/kg b.wt) increased the life span of tumor-bearing animals by merely 23.99%, but significantly different from the survival rate of untreated animals. Similar results of anti-tumor activity in EAC-induced tumors were previously reported by Samudrala, et al. [39] with *Alternanthera brasiliana,* and Senthil Kumar et al. [40], with *Prosopis glandulosa*. In EAC-induced models, the survival times of mice and the final volumes of the tumors were positively correlated, revealing the progressive growth pattern. Tumor growth is also enhanced by the amount of ascitic fluid in the mouse peritoneal cavity, which is an inflammatory exudate produced in response to EAC cell growth [41,42]. In all EAC-induced animals of our study, there was a gradual increase noticed in ascites fluid volume, pertinent to the above concept. 

Solid tumors (e.g., sarcomas, carcinomas, and lymphomas) are abnormal, localized masses of tissues devoid of cysts or liquid areas, of either benign or malignant nature [43,44]. Dalton’s lymphoma spontaneously originates in the thymus of murine animals in the transplantable form [45,46,47] and, hence, widely used in cancer research, with tumor volume measurement as a reproducible parameter. The current study also revealed that oral administration of OIM extract brought about a gradual, but significant reduction in tumor volume, which was evident from the 12th day of induction onwards up to day 30, signifying the anticancer property of the extract, consistent with previous anticancer studies of plant extracts [48,49] using DLA induced tumors.

The study reveals the significant anti-carcinogenic efficacy of the root bark extract of *O. indicum* Vent. in suppressing papilloma genesis, which is induced by the application of DMBA and croton oil. In the typical two-stage chemical carcinogenesis system in the mouse skin, a mutation in *Hras1* induced by a low dose of 7,12-dimethyl benz(a)anthracene is promoted by repeated application of TPA, which acts as a tumor promoter. During the tumor promotion stage, due to the altered expression of genes whose encoded protein products take part in hyperproliferation, tissue remodeling, and inflammation, the initiated cells are subjected to selective clonal expansion, forming visible tumors [50]. This facilitates the initiation and promotion stages to be distinctly viewed both operationally and mechanistically [51]. 

The inhibition of tumorigenesis, as revealed from the study, has occurred in the promotion stage. Topical application of OIM extract showed a dose-dependent inhibition of skin tumorigenesis, with a delay of three and six weeks respectively, in high and low-dose groups, compared to the full-blown skin tumors that developed in control mice, having received no external treatment. Chemoprevention, as stated by Wattenberg [52], can occur either during initiation or during a promotion; the mediators preventing the former are categorized as blocking agents, and those inhibiting the latter as suppressing agents. Chemoprevention can be best accomplished during the promotion stage of carcinogenesis because of its reversible nature which requires more time and a higher incidence of exposure, unlike initiation or progression [53,54].

The relationship between inflammation and papilloma-genesis has been previously established. During human papillomavirus infection, the host inflammatory response is postulated to promote lesion progression through the direct participation of inflammatory cells. Under the influence of the microbial genome or epigenetic factors, somatic cells undergo changes mediated by autocrine and paracrine signals indicative of the association between chronic inflammation and cancer [55,56]. In addition to being a component of *Dashamoola* formulation, there is also mention of the usage of *O. indicum* Vent. as a single ingredient in treating rheumatoid arthritis and inflammation [57,58]. The methanolic extract root bark of *O. indicum* Vent. has been previously reported by the authors indicating its potential anti-inflammatory properties in acute and chronic paw edema models [59] in mice. In light of the above facts, it is conclusive that the inhibitive action of OIM extract may be due to its suppressive effect on inflammation. 

In the context of serious side effects posed by chemotherapeutic agents used in cancer therapy, a surge has been observed in developing complementary medication from plant sources, which are repositories of bioactive compounds. In the antitumor study conducted by us, cyclophosphamide was used to treat the positive control group, the compound being a common chemotherapeutic agent [60], which is also known to cause myelosuppression and anemia [61]. In our study, it was observed that, though cyclophosphamide treatment increased the average life span in ascites tumor models and reduced solid tumors, the animals developed anemia and suffered a decrease in total leucocyte count, as revealed from the hematological examination. Contrarily, the study reveals that OIM extract exerts simultaneous antitumor and myeloprotective activities, similar to other plant extracts rich in bioactive compounds [62,63], possibly due to the synergistic effect of these substances present in them. 

The root bark of *O. indicum* has been found to contain chrysin, baicalein, biochanin-A (flavonoids), and ellagic acid (phenolic compound) [38]. With previously reported anticancer activities of baicalein [64,65], chrysin [66,67], biochanin-A [68], and ellagic acid [69], it may be concluded that the antitumor activity of the extract is due to the combined activity of these bioactive components, exerting anti-carcinogenic and cytotoxic effects.

## 4. Materials and Methods

### 4.1. Reagents and Chemicals

7,12-dimethyl-benz[a] anthracene of an analytical grade was used for the study (Sigma Aldrich, MO, USA); the hematological test kit manufactured by Agappe Diagnostics (Ernakulam, Kerala, India) was used for hemoglobin estimation. The reagents and chemicals that were used in the study were of analytic grade.

### 4.2. Collection of Oroxylum Indicum and Methanol Extraction

The roots of *Oroxylum indicum* Vent. were collected from Thrissur and authenticated (KFRI/SILVA/GEN/06/11). The peeled bark was dried, powdered, and extracted using methanol as solvent. The methanol extract was selected based on preliminary studies and also considering the previous studies that methanol extracts most of the phytochemicals [70,71]. The concentrated extract was dissolved in the deionized water for the animal model studies and cytotoxicity analysis.

### 4.3. UPLC-Q-TOF-MS Analysis

Ultra-high-pressure liquid chromatographic (UPLC) analysis of the extract was performed using an Acquity UPLC H class (Waters) system equipped with an autosampler and a diode-array detector (DAD). The mobile phase (methanol and 0.1% aqueous formic acid) was allowed to percolate through a BEH C18 column (with specifications, 50 mm × 2.1 mm × 1.7 μm, purchased from Waters, USA) using gradient elution (0–5 min, 5% acetonitrile; 5–7 min, 95% methanol; 8–9 min 5% methanol) at a flow rate of 0.3 mL/min. Detection was achieved at a wavelength of 210–400 nm. The MS and MS/MS data were retrieved from Xevo G2 (Waters, Massachusetts, USA) Quadrupole-Time-of-Flight (Q-TOF) system. Mass spectrometric operations were carried out by the methods of Felipe, et al. [72].

The quantification of flavonoids chrysin and baicalein were also carried out using the same solvent and running conditions using HPLC SCL-10A VP (Shimadzu, Kyoto, Japan). The standard compounds were initially dissolved in 0.1 mg/mL concentration and loaded in the HPLC system. Similarly, the extract was analyzed and compared with the standards, and the final concentration was estimated using SCL-VP software.

### 4.4. Animals

BALB/c mice weighing 20–25 g were grouped and the in vivo acute toxicity analysis of *O. indicum* Vent. root bark extract was conducted for 14 days according to OECD guideline-423 [73]. Based on the acute toxicity studies conducted by the authors as per OECD—423 guidelines, OIM extract was found to be having no observed adverse effect limit (NOAEL) till 5 g/ kg [59]. For further studies, 400 mg/kg b.wt. of OIM was selected as the high dose (OIMH) and 200 mg/kg of OIM as the low dose (OIML). 

### 4.5. Cell Lines

Dalton’s lymphoma ascites tumor cells (DLA) and Ehrlich ascites carcinoma (EAC) cells were aspirated aseptically in PBS (0.1 M, pH 7.4). The wash was repeated thrice and the cell count was set as 10^7^ cells/mL and used for cytotoxicity and antitumor studies [74]. 

### 4.6. Cytotoxic Analysis of O. indicum on DLA and EAC Cells

About 0.1 mL of the DLA/EAC cell suspension was dispensed into test tubes containing OIM extract, at concentrations ranging from 100 to 500 µg/mL. After the incubation for 3 h, cell viability was estimated by trypan blue exclusion [75]. The half-maximal cell death (IC_50_) was also estimated graphically. Spleen cells isolated from BALB/c mice were used as normal [76]. 

### 4.7. Antitumor Study

#### 4.7.1. Ascites Tumor Model

The ascites tumor was induced in BALB/c mice by intra-peritoneal injection of 1 × 10^6^ EAC cells/animal. Animals were divided into four groups, each with 6 members. Group I served as the untreated negative control. Group II served as the standard or positive control group treated with cyclophosphamide (i.p.) at a dosage of 10 mg/kg b.wt. Groups III and IV were the extract-treated groups receiving oral administration of methanol extract of *O. indicum* Vent. root bark (OIM) extract at low (OIML—200 mg/kg b.w.) and high doses (OIMH—400 mg/kg b.w.), respectively. The drug treatment was conducted for 10 days after 24 h of induction and the percentage increase in lifespan was calculated [77]. 

#### 4.7.2. Solid Tumor Model

To induce a solid tumor, the intramuscular injection was given to the hind limb with 1 × 10^6^ cells/animal. The grouping of animals and drug treatment protocol followed was the same as that of the ascites tumor model. The tumor development was measured using vernier calipers every 3rd day after induction, up to day 30) and the volume was calculated [78].

### 4.8. Hematological Parameters

The total WBC count and hemoglobin level of the animals in the solid tumor study were tracked every third day to compare the effect of cyclophosphamide and OIM extract on hematological parameters. The caudal vein blood was drawn into heparinized tubes and the total WBC count was determined by a standard procedure using the hemocytometer [79] and the hemoglobin level was estimated using a standard kit procedure (cyan methemoglobin method) (Agappe Diagnostics Kit).

### 4.9. DMBA–Croton Oil-Induced Papilloma

The study was conducted using male BALB/c mice. Three animals were accommodated per cage to avoid fighting and skin aberrations that may result; aggressive members were maintained separately. A circular area of diameter 2 cm on the dorsal side of each mouse was shaved. These mice were grouped as followed (*n* = 6);

Group I: 470 nmol of DMBA /mouse in 200 μL acetone as a single dose on day 1.

Group II: 1% croton oil/mouse in 200 μL acetone, (2 weeks after DMBA application, in a frequency of two times per week for 6 continuous weeks).

Group III: DMBA (same regime as for group I) plus croton oil (same regime as for group II). This group served as a control.

Group IV and V: [(DMBA + croton oil—same regime as for group III)] + *Oroxylum indicum* Vent. methanolic extract low dose (OIML)—5% in 200 μL distilled water and high dose (OIMH)—10% in 200 μL, respectively, 30 min before each croton oil application

Throughout the study period of 20 weeks, all animals were monitored for their food intake and toxicity symptoms. Weekly formation of papillomas, the percentage incidences of papillomas, the cumulative count of papillomas developed, the number of papillomas developed per animal, and the delay in tumor onset in each group were noted [80].

### 4.10. Statistical Analysis

The data representation followed a mean ± SD format with six animals per group. The comparison was done using one-way ANOVA followed by the Dunnett post hoc test using Graph Pad Prism 7.0.

## 5. Conclusions

The study concludes that the root bark extract of *O. indicum* is a rich source of various flavonoid compounds that are capable of inducing strong anticancer properties. In addition, the extract improved the life expectancy and tumor burden in the mice without inducing toxic insults, such as what occurs in cytotoxic anticancer chemotherapeutic agents.

## Figures and Tables

**Table 1:** The compounds tentatively identified from *O. indicum* Vent. wild root bark (OIM) extract by UPLC-Q-TOF-MS analysis.

Peak No.	RT (Min)	*m/z*	Molecular Weight (kDa)	Molecular Formula	Name of the Compound
1	0.79	562.2056	−	−	Unidentified
2	2.92	487.1551	−	−	Unidentified
3	3.70	639.2048	−	−	Unidentified
4	3.90	637.1891	638.184685	C_29_H_34_O_16_	Demethoxycentaureidin 7-*O*-rutinoside
5	4.02	653.2208	−	−	Unidentified
6	4.13	623.2098	624.16903	C_28_H_32_O_16_	Isorhamnetin-3-*O*-rutinoside (Narcissin)
7	4.36	607.2140	−	−	Unidentified
8	4.52	547.1558	−	−	Unidentified
9	4.75	651.2419	−	−	Unidentified
10	4.99	445.1238	446.08491	C_21_H_18_O_11_	Baicalein-7-*O*-glucuronide (Baicalin)
11	5.65	269.0502	270.05282	C_15_H_10_O_5_	5,6,7-Trihydroxyflavone (Baicalein)
12	5.82	327.2242	328.094688	C_18_H_16_O_6_	3-Hydroxy-3′,4′,5′-trimethoxyflavone
13	6.02	283.0659	284.068473	C_16_H_12_O_5_	5,7-Dihydroxy-3-(4-methoxyphenyl)chromen-4-one (Biochanin A)
14	7.05	299.2069	300.099774	C_17_H_16_O_5_	4′-Hydroxy-5,7-dimethoxyflavanone
15	7.23	295.2332	296.104859	C_18_H_16_O_4_	6-Ethoxy-3(4′-hydroxyphenyl)-4-methylcoumarin
16	7.80	311.1749	−	−	Unidentified
17	8.16	325.1904	−	−	Unidentified

**Table 2:** The concentration of baicalein (Bcl) and chrysin (Chr) expressed as mg/g dry wt. of powder in *O. indicum* Vent. medium.

Compound	Concentration
Baicalein	1.794 ± 0.23
Chrysin	0.725 ± 0.08

## Data Availability

Data can be made available upon valid request.

## Supplementary Materials

The following supporting information can be downloaded at: https://www.mdpi.com/article/10.3390/molecules27238459/s1, Figure S1: HPLC chromatograms of wild extract and the flavonoids—baicalein (Bcl) and chrysin (Chr).
