# Supplementary material for: Root Bark Extract of Oroxylum indicum Vent. Inhibits Solid and Ascites Tumors and Prevents the Development of DMBA-Induced Skin Papilloma Formation
Source: Molecules. 2022 Dec 2;27(23):8459. doi: 10.3390/molecules27238459 (PMC9738881; doi:10.3390/molecules27238459)
Supplement: Supplementary file 1 [file molecules-27-08459-s001.zip › molecules-2046556-supplementary.pdf]

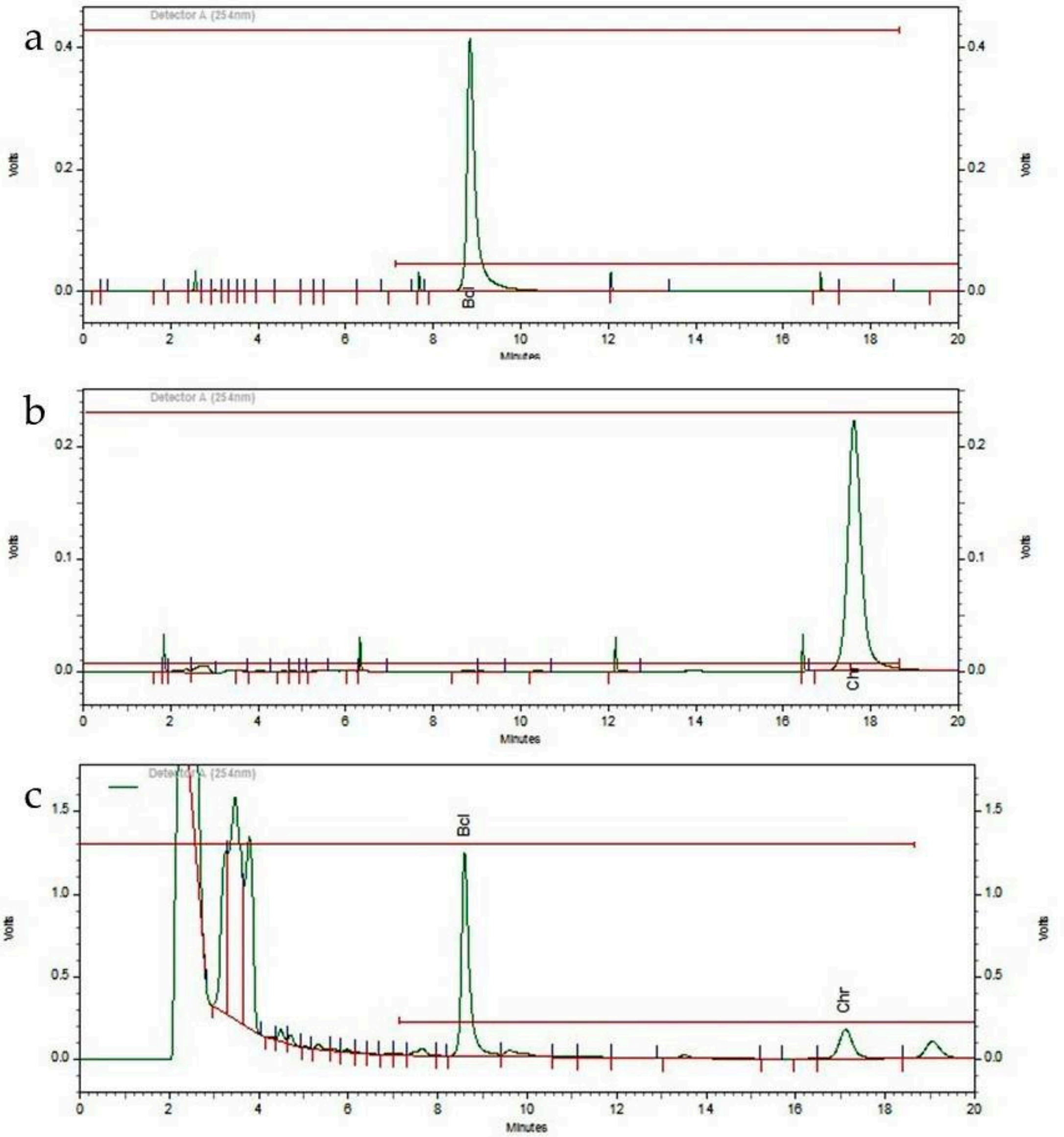

Supplementary Figure S1. HPLC chromatograms of wild extract and the flavonoids – baicalein (Bcl) and chrysin (Chr)
